# Effects of pulmonary rehabilitation on ventilation dynamics measured during exertion in patients with post-acute COVID-19 syndrome: A cross-sectional observational study

**DOI:** 10.1371/journal.pone.0296707

**Published:** 2024-02-02

**Authors:** Renan Pereira Campos, Jéssica Gabriela Messias Oliveira, Iasmim de Oliveira Farias, Viviane Cristina Viana de Souza, Samantha Gomes de Alegria, Rosemere Saldanha Xavier, Agnaldo José Lopes

**Affiliations:** 1 Rehabilitation Sciences Post-Graduation Programme, Augusto Motta University Centre (UNISUAM), Rio de Janeiro, Brazil; 2 Pos-Graduation Programme in Medical Sciences, State University of Rio de Janeiro (UERJ), Rio de Janeiro, Brazil; 3 Faculty of Physiotherapy, Augusto Motta University Centre (UNISUAM), Rio de Janeiro, Brazil; 4 Local Development Post-Graduation Programme, Augusto Motta University Center (UNISUAM), Rio de Janeiro, Brazil; University of Chichester - Bishop Otter Campus: University of Chichester, UNITED KINGDOM

## Abstract

**Background:**

Pulmonary rehabilitation (PR) is recommended in people with post-acute COVID-19 syndrome (PACS), although there is a lack of studies evaluating its benefits via the most commonly used primary endpoint: the six-minute walk test (6MWT). This study evaluated the effects of PR on the dynamics of ventilation measured during the 6MWT in patients with PACS and, secondarily, evaluated the association of these findings with measures of lung function and structure.

**Methods:**

This was an observational cross-sectional study of patients with PACS, in which 33 had undergone PR (PR-PACS group) and 32 had not undergone PR (NPR-PACS group). These patients underwent Spiropalm®-equipped 6MWT with measurement of inspiratory capacity (IC) to evaluate dynamic hyperinflation (DH). In addition, they performed spirometry, impulse oscillometry (IOS) and lung ultrasound (LUS).

**Results:**

Spirometry was abnormal in 21.2% and 31.3% of participants in the PR-PACS and NPR-PACS groups, respectively (p = 0.36). IOS was abnormal in 28.6% and 66.7% of participants in the PR-PACS and NPR-PACS groups, respectively (p = 0.003). LUS was altered in 39.4% and 43.8% of the participants in the PR-PACS and NPR-PACS groups, respectively (p = 0.72). The 6-min walk distance (6MWD) was greater in the PR-PACS group than in the NPR-PACS group (p = 0.001]. HD was observed in 6.1% and 37.5% of participants in the PR-PACS and NPR-PACS groups, respectively, with a significant difference in ΔIC (p<0.001). The 6MWD correlated significantly with several IOS parameters and with ΔIC.

**Conclusions:**

Patients with PACS undergoing PR perform better in the 6MWT, with a higher 6MWD and less HD. In these patients, IOS is able to distinguish the effects of PR that are not differentiated by spirometry or LUS. Furthermore, the better the respiratory mechanics assessed by IOS and the less DH there was, the higher the performance in the 6MWT.

## Introduction

Regardless of the severity of the disease or the need for hospitalization, patients who have had COVID-19 are at high risk of presenting one or more sequelae or new symptoms 3 months after acute infection, a condition recognized as post-acute COVID-19 syndrome (PACS) [1]. The prevalence of patients who present at least one residual symptom after recovery from the acute event ranges from 43% to 62% [2]. It is believed that persistent inflammation and deconditioning may contribute to systemic complications in PACS, which supports the need for a more in-depth assessment of functional capacity during exercise [3]. In fact, persistence of dyspnea is common in PACS, even after the normalization of pulmonary function tests (PFTs) [4, 5]. In this sense, it is important to understand the impact of the disease on the functional capacity during exercise in PACS in the long term, as well as the impact of pulmonary rehabilitation (PR) [6].

The lungs are the organ most affected by COVID-19, and respiratory symptoms and exercise intolerance are prevalent [7]. The persistence of dyspnea in patients who had pneumonia or milder forms of COVID-19 does not seem to be associated with the degree of disease severity or residual impairment of lung function [8]. Post-COVID-19 dyspnea and exercise intolerance are also potential contributors to small airway dysfunction and lung hyperinflation, which are demonstrated in these individuals [9–12]. Notably, traditional PFTs, although informative, may not capture all post-COVID-19 functional and structural changes, including those resulting from a PR program [12]. Although the lungs are the main target organ for rehabilitative strategies in individuals with PACS, the results of PR on lung function are inconsistent [13]. While one study did not detect any effect of rehabilitation on PFTs [10], another study reported a significant improvement in all parameters investigated after respiratory muscle training [14].

PACS can be debilitating, with nearly half of cases requiring a reduction in workload [12]. In fact, these people experience exercise intolerance, impaired daily function and low quality of life (QoL) and should be directed to PR. Using cardiopulmonary exercise testing (CPET), a study suggested that reduced operating volumes are the main determinant of dyspnea in these patients, and decreased alveolar volume reflects the heterogeneity of residual lung damage after the acute phase of COVID-19 [15]. In fact, the magnitude of the effects of PR on lung function and exercise tolerance of patients with PACS is still a matter of debate, as it may be dependent on the operant mechanisms of exercise limitations and the rehabilitative strategies employed [16, 17]. Although there are effects on dyspnea and physical capacity, the certainty of the evidence of physical training programs and breathing exercises in patients with PACS is low, which means that there is still much uncertainty about these effects [18].

Ventilatory limitation during exercise is generally assessed by the breathing reserve (BR), which indicates how closely the minute ventilation (VE) approximates the ventilatory capacity during a given activity [19]. Another measure to assess ventilatory limitation is dynamic hyperinflation (DH), which refers to exercise-induced air trapping and is a major contributor to exercise intolerance in patients with lung diseases. As the respiratory rate increases during exertion, the expiratory time decreases, and the end expiratory lung volume and inspiratory capacity (IC) increase [20]. In patients with PACS, ventilatory inefficiency during exercise with increased dead space ventilation is common, even in those with peak oxygen uptake (VO_2peak_) is normal [5]. Although VO_2peak_ is reduced, there seem to be no differences in ventilation, BR and ventilatory efficiency among those who required and did not require treatment in an intensive care unit (ICU) [21]. Based on the assumptions found in studies involving CPET, the approach of associating dynamic ventilation measures during submaximal exercise in individuals with PACS may help to understand the mechanisms of exercise limitation in this population and, therefore, increase the recommendations for PR.

Although PR has been recommended in PACS, there is a lack of formal studies that thoroughly evaluate its effects. The primary endpoint to assess the benefits of PR is the measurement of exercise capacity [22]. Using CPET, some studies observed both ventilatory and circulatory limitations [5, 7], although the effects of PR on the dynamics of ventilation have not been reported. Although CPET is considered the gold standard for assessing exercise capacity and dyspnea on exertion, its high cost and lack of available technical apparatus limit its use in the tremendous number of patients with PACS. An alternative is the 6-minute walk test (6MWT), where better performance illustrates muscular, cardiac and respiratory adaptations and the 6-min walk distance (6MWD) is a reliable indicator of physical and respiratory adaptations in patients with lung disease [23]. In this context, Spiropalm^®^-equipped 6MWT is an important clinical tool that provides assessment of ventilatory responses to exercise that are not adequately reflected by measurement of resting lung function. At PACS, we hypothesized that the Spiropalm®-equipped 6MWT may be an approach to unmask functional abnormalities in the respiratory system reversed by PR. Thus, the present study aimed to evaluate the effects of PR on the dynamics of ventilation measured during the 6MWT in patients with PACS.

## Methods

### Patients

Between August 2023 and October 2023, patients with PACS aged ≥18 years who were seen at the Piquet Carneiro Policlinic, State University of Rio de Janeiro, Rio de Janeiro, Brazil were enrolled in this cross-sectional observational study. Inclusion criteria were patients with a history of COVID-19 pneumonia and the continuation or the development of new symptoms 3 months after the initial infection, which lasted for at least 2 months without other explanation per the World Health Organization specifications [1] and who had not been hospitalized at the time of the acute infection. The following exclusion criteria were adopted: absence of a previous diagnosis of COVID-19 confirmed by reverse transcriptase polymerase chain reaction (RT‒PCR), report of acute respiratory failure related to COVID-19, presence of musculoskeletal disorders and patients who were unable to undergo the protocol tests. For comparative purposes, the participants were divided into two groups according to whether they had performed prior PR at the outpatient level completed up to 1 month before the evaluations: participants who had undergone PR (PR-PACS group) and participants who had not undergone PR (NPR-PACS group).

The project was approved by the Augusto Motta University Center, Rio de Janeiro, Brazil, under protocol number CAAE-50700921.5.0000.5235. The protocol was registered with the ClinicalTrials.gov identifier code NCT05967039. Written informed consent and verbal consent prior to enrolment was mandatory. Anonymous personal identifiers were used for each participant.

### Procedures

Information on patient demographics, such as sex, age, weight, height, body mass index (BMI), smoking status, physical activity, and comorbidities, and COVID-19-related information, such as (RT-PCR test result, severity, and time since diagnosis of COVID-19, was collected in face-to-face interviews by the physical therapist. Patients who had undergone PR completed individualized resistance, strength and inspiratory muscle training over a period of 8 weeks, 2–3 times a week for 3–4 hours each, under the supervision of physicians and physical therapists.

### Measurements and outcomes

A Post-COVID-19 Functional Status (PCFS) scale was applied following the instructions provided in the primary source [24]. Briefly, the participants were asked about their average situation in the previous week regarding symptoms (for example, dyspnea, pain, fatigue, muscle weakness, memory loss, depression and anxiety). The meaning of each of the PCFS scale scores is as follows: grade 0 (no functional limitations), grade 1 (negligible functional limitations), grade 2 (slight functional limitations), grade 3 (moderate functional limitations), and grade 4 (severe functional limitations) [25].

The spirometric test was performed in Vitatrace VT 130 SL equipment (Codax Ltda, Rio de Janeiro, Brazil), following previous standards [26] and using reference values from Pereira et al. [27] Restrictive damage was suggested by forced vital capacity (FVC) <80% predicted and forced expiratory volume in 1 sec over FVC (FEV_1_/FVC) ≥70% predicted, while obstructive damage was defined by FEV_1_/FVC <70% predicted [28]. Additionally, we also performed impulse oscillometry (IOS) using Quark i2m equipment (Cosmed, Rome, Italy) following previous standards [29]. The following resistive and reactive parameters were evaluated: respiratory system resistance (Rrs) at 4 Hz (R4) and 20 Hz (R20), mean resistance between 4–20 Hz (Rm), heterogeneity of resistance between 4–20 Hz (R4-R20), resonance frequency (Fres), and area under the reactance curve (AX). The following values were considered abnormal: R4 and/or R20 ≥150% of predicted, Fres >12 Hz, AX >3.60 cm H_2_O/L/s, and R4-R20 >20%, which was also used for the diagnosis of small airway disease (SAD) [30, 31].

Lung ultrasound (LUS) was performed on Aplio XG equipment (Toshiba Medical Systems, Tokyo, Japan) coupled to a 7.5–10 MHz linear multifrequency transducer or to a 3.5–5 MHz convex transducer in B mode. The exams were performed by pulmonologists, and all of them had at least 9 years of experience in LU. All evaluations of LUS were performed by 2 examiners, and when there was disagreement between them, a consensus was reached. With the participants in the sitting position, the capture of LUS signs was performed in 6 areas of each hemithorax as follows: anterior, two lateral and two posterior [32]. In the evaluation of the signs of LUS, we sought to find B-lines >2, coalescent B-lines and subpleural consolidations. To obtain the aeration score, points were assigned to each of the 6 areas as follows: B-lines >2, 1 point; coalescent B-lines, 2 points; and consolidations, 3 points. Thus, the sum of all areas represented the aeration score [33].

The 6MWT was performed according to previous recommendations [21]. Additionally, a handheld silicone face mask (Spiropalm 6MWT, Cosmed, Rome, Italy) was attached to the patient. Before the test, the IC measurement was obtained while the patient was seated in the chair located at the starting line of the 30-meter course. To perform the test, participants were instructed to walk as many times as possible for 6 minutes on a flat stretch of hard surface indoors, marked on the ground with cones at both ends. The 6MWT was preceded and followed by measurement of blood pressure, heart rate (HR), respiratory rate and peripheral oxygen saturation (SpO_2_). The 6MWT was interrupted if SpO_2_ <80%, exhaustion, chest pain or intolerable cramps. Oxygen desaturation was defined as a decrease in SpO_2_ ≥4% [34]. DH was then measured again at the end of the 6MWT with the same procedure, and a ≥ 100 mL decrease in IC (ΔIC) during exercise was defined as DH [20]. In addition to DH, other dynamic ventilatory responses were measured, including VE and BR. The latter was calculated as the difference between maximal voluntary ventilation (MVV) and VE_peak_ ([MVV-VE_peak_]/MVV); BR < 30% was considered ventilatory limitation on exertion [35]. MVV was determined automatically by the device as FEV_1_ multiplied by 40. The 6MWT was performed in duplicate with a 30-minute interval between them. The highest 6MWD was compared to the values reported by Britto et al. [36], and it was considered abnormal if it was < 80% of the predicted value.

### Statistical analysis

The normality of the data distribution was assessed using the Shapiro–Wilk test and graphical analysis of the histograms. The data were expressed as measures of central tendency and dispersion suitable for numerical data and as frequency and percentage for categorical data. The comparison between the groups PR-PACS and NPR-PACS were analyzed using Student’s *t* test for independent samples (or Mann–Whitney test) for numerical variables and the chi-square test (or Fisher’s exact test) for categorical variables. The association between the 6MWD and all the variables studied was analyzed using Pearson’s correlation coefficient (or Spearman’s) for numerical data and Student’s *t* test for independent samples for categorical data. The relative delta of IC was calculated as follows: ΔIC (%) = (final IC–initial IC)/initial IC x 100). Statistical analysis was performed using IBM SPSS Statistics version 26.0 software (IBM Corp., Armonk, NY, USA).

## Results

Among the 71 patients eligible for the study, five were excluded for the following reasons: musculoskeletal disorders preventing the performance of the 6MWT (n = 3) and a history of acute respiratory failure at the time of COVID-19 infection (n = 2). Of the 65 PACS patients included in the study, 33 were in the PR-PACS group, and 32 were in the NPR-PACS group. The mean age was 41.3 ± 10.6 years and 45.2 ± 10.9 years (p = 0.37) in the PR-PACS and NPR-PACS groups, respectively, while the mean BMI was 29.8 ± 5.1 and 32 ± 5.2 kg/m^2^ in the PR-PACS and NPR-PACS groups, respectively (p = 0.094). The average time since diagnosis of COVID-19 was 7.1 ± 1.8 and 8.8 ± 1.9 months in the PR-PACS and NPR-PACS groups, respectively (p = 0.35). Regarding smoking, 6 (18.2%) and 4 (12.5%) were former or current smokers in the PR-PACS and NPR-PACS groups, respectively (p = 0.39). Physical activity was reported by 13 (39.4%) and 8 (25%) participants in the PR-PACS and NPR-PACS groups, respectively (p = 0.21). Comparisons between the two groups regarding demographic data and comorbidities are shown in Table 1.

**Table 1:** Comparisons of demographic data and clinical findings between patients who did and did not undergo pulmonary rehabilitation.

Variable	Total sample (n = 65)	PR-PACS group (n = 33)	NPR-PACS group (n = 32)	p-value*
**Demographic data**				
Male/female	19/46	9/24	10/22	0.41
Age (years)	43.1 ± 10.7	41.3 ± 10.6	45.2 ± 10.9	0.37
Body mass (kg)	83.6 ± 17	81.6 ± 14.3	85.8 ± 19.4	0.32
Height (m)	1.65 ± 0.10	1.65 ± 0.07	1.64 ± 0.12	0.44
BMI (kg/m^2^)	30.9 ± 5.2	29.8 ± 5.1	32 ± 5.2	0.09
**Comorbidities, n (%)**				
Hypertension	22 (33.8)	9 (27.3)	13 (40.6)	0.26
Diabetes	16 (20)	6 (18.2)	10 (31.2)	0.28
Cardiopathy	8 (12.3)	4 (12.1)	4 (12.5)	0.63
Asthma	8 (12.3)	6 (18.2)	2 (6.3)	0.14
COPD	5 (7.7)	2 (6.1)	3 (9.4)	0.49
**PCFS scale, n (%)**				
0–1	46 (70.8)	27 (81.8)	19 (59.4)	**0.046**
2–4	19 (29.2)	6 (18.2)	13 (40.6)	

Data are given as mean ± SD or number (%). The value in bold refer to significant difference.

* Difference between PR-PACS and NPR-PACS groups. PR-PACS = patients with post-COVID syndrome who underwent pulmonary rehabilitation; NPR-PACS = patients with post-COVID syndrome who did not undergo pulmonary rehabilitation; COPD = chronic obstructive pulmonary disease; PCFS = Post-COVID-19 Functional Status.

Spirometry was abnormal in 7 (21.2%) and 10 (31.3%) participants in the PR-PACS and NPR-PACS groups, respectively, with no significant difference between them (p = 0.36). The PR-PACS group showed normal function, restrictive damage and obstructive damage in 26 (78.8%), 6 (18.2%) and 1 (3%) participant, respectively, while the NPR-PACS group showed normal function, restrictive damage and obstructive pulmonary disease in 22 (68.8%), 8 (25%) and 2 (6.3%) participants, respectively. The IOS was abnormal in 8 (28.6%) and 20 (66.7%) participants in the PR-PACS and NPR-PACS groups, respectively, with a significant difference between them (p = 0.003). In IOS, abnormal Fres was observed in 13 (39.4%) and 17 (53.1%) participants, while abnormal AX was observed in 8 (24.2%) and 12 (37.5%) participants of the PR-PACS and NPR-PACS groups, respectively. At IOS, the diagnosis of PAS was observed in 4 (14.6%) and 9 (30%) participants in the PR-PACS and NPR-PACS groups, respectively, with no significant difference between them (p = 0.15).

Regarding the LUS, the test was abnormal in 13 (39.4%) and 14 (43.8%) participants in the PR-PACS and NPR-PACS groups, respectively, with no significant difference between them (p = 0.72). The median aeration score was 0 (0–1) and 0 (0–2) points in the participants in the PR-PACS and NPR-PACS groups, respectively, with no significant difference between them (p = 0.09). Comparisons between the two groups regarding pulmonary function parameters and LUS signs are shown in Table 2.

**Table 2:** Comparisons of pulmonary function parameters and lung ultrasound signals between patients who did and did not undergo pulmonary rehabilitation.

Variable	Total sample (n = 65)	PR-PACS group (n = 33)	NPR-PACS group (n = 32)	p-value*
**Spirometry**				
FVC (% predicted)	88.7 ± 11	88.5 ± 8.6	88.9 ± 13.2	0.88
FEV_1_ (% predicted)	87.7 ± 12.3	87.2 ± 10.9	88.1 ± 13.8	0.77
FEV_1_/FVC (%)	84.1 ± 7	86.3 ± 7.4	81.9 ± 6.1	**0.011**
FEF_25-75%_ (% predicted)	92.8 ± 26.8	93.5 ± 17.8	92.1 ± 34	0.83
**Impulse oscillometry**				
Fres (Hz)	13 (10–20)	11 (9–19)	17 (11–21)	**0.041**
Rm (cm H_2_O/L/s)	5.4 (3.2–6)	5.4 (3.2–6.7)	5.3 (3.2–6.2)	0.64
R4 (% predicted)	137 (126–164)	134 (118–155)	141 (126–168)	0.48
R20 (% predicted)	142 (118–169)	139 (118–139)	151 (117–178)	0.41
R4-R20 (cm H_2_O/L/s)	0.7 (0.3–1.6)	0.6 (0.4–1.5)	0.8 (0.3–2)	0.64
AX (cm H_2_O/L)	3.1 (0.8–4.8)	2.5 (0.3–4)	3.8 (1.8–5.1)	**0.033**
**Lung ultrasound**				
B-lines >2, n (%)	23 (35.4)	9 (27.3)	14 (43.8)	0.16
Coalescent B-lines, n (%)	9 (13.8)	6 (18.2)	3 (9.4)	0.25
Subpleural consolidations, n (%)	8 (12.3)	6 (18.2)	2 (6.3)	0.14
Aeration score	0 (0–1)	0 (0–1)	0 (0–2)	0.09

Data are given as mean ± SD, median (IQR) or number (%). The values in bold refer to significant differences.

* Difference between PR-PACS and NPR-PACS groups. PR-PACS = patients with post-COVID syndrome who underwent pulmonary rehabilitation; NPR-PACS = patients with post-COVID syndrome who did not undergo pulmonary rehabilitation; FEF_25-75%_ = forced expiratory flow during the middle half of the FVC maneuver; Fres = resonance frequency; Rm = mean resistance between 4–20 Hz; R4, resistance at 4 Hz; R20, resistance at 20 Hz; R4-R20, heterogeneity of resistance between R4 and R20; AX, area under the reactance curve.

Participants in the PR-PACS group walked more in the 6MWT than participants in the NPR-PACS group [437 ± 92 vs. 361 ± 79 m, p = 0.001]. Fifteen (45.5%) and 20 (62.5%) participants in the PR-PACS and NPR-PACS groups, respectively, had a predicted 6MWD <80%. One (3%) and 3 (9.4%) participants in the PR-PACS and NPR-PACS groups, respectively, desaturated during the 6MWT. DH was observed in 2 (6.1%) and 12 (37.5%) participants in the PR-PACS and NPR-PACS groups, respectively. BR depletion was not observed in any participant in the PR-PACS group, although it was detected in 4 (12.5%) participants in the NPR-PACS group. Comparisons between the two groups regarding performance during the Spiropalm^®^-equipped 6MWT are shown in Table 3.

**Table 3:** Performance during the spiropalm^®^-equipped six‐minute walk test between patients who did and did not undergo pulmonary rehabilitation.

Variable	Total sample (n = 65)	PR-PACS group (n = 33)	NPR-PACS group (n = 32)	p-value*
6MWD (m)	400 ± 94	437 ± 92	361 ± 79	**0.001**
6MWD (% predicted)	77.6 ± 17.7	83 ± 17.2	72.2 ± 16.6	**0.012**
Basal SpO_2_ (%)	97 (96–98)	96 (96–98)	96 (95–98)	0.20
End-of-test SpO_2_ (%)	96 (95–97)	96 (95–98)	96 (94–97)	0.075
Basal HR (pulse/min)	86 (75–91)	77 (70–88)	88 (83–94)	**<0.001**
End-of-test HR (pulse/min)	108 (93–114)	105 (91–110)	110 (96–115)	0.077
Resting VE (L/min)	7.9 (6.7–10)	8.2 (7.1–10)	7.4 (6–10)	0.17
VE_peak_ (L/min)	19 (15–27)	17 (14–22)	20 (16–29)	0.10
BR, median (%)	71 (68–80)	71.9 (70–81)	70 (64–79)	0.24
Basal IC (L)	2.1 (1.8–2.4)	1.9 (1.6–2.3)	2.2 (1.9–2.5)	**0.047**
End-of-test IC (L)	2.3 (1.9–2.6)	2.3 (2.1–2.6)	2.2 (1.8–2.5)	0.072
ΔIC (L)	0.2 (0–0.5)	0.4 (0.2–0.6)	0 (-0.1–0.2)	**<0.001**
ΔIC (%)	14.3 (4.8–32)	27.8 (16.4–46)	7.1 (-6.9–14)	**<0.001**

Data are given as mean ± SD or median (interquartile range). The values in bold refer to significant differences.

* Difference between PR-PACS and NPR-PACS groups. PR-PACS = patients with post-COVID syndrome who underwent pulmonary rehabilitation; NPR-PACS = patients with post-COVID syndrome who did not undergo pulmonary rehabilitation; 6MWD = six‐minute walking distance; SpO_2_ = oxygen saturation; HR = heart rate; VE = minute ventilation; BR = breathing reserve; IC = inspiratory capacity.

**Fig 1:**
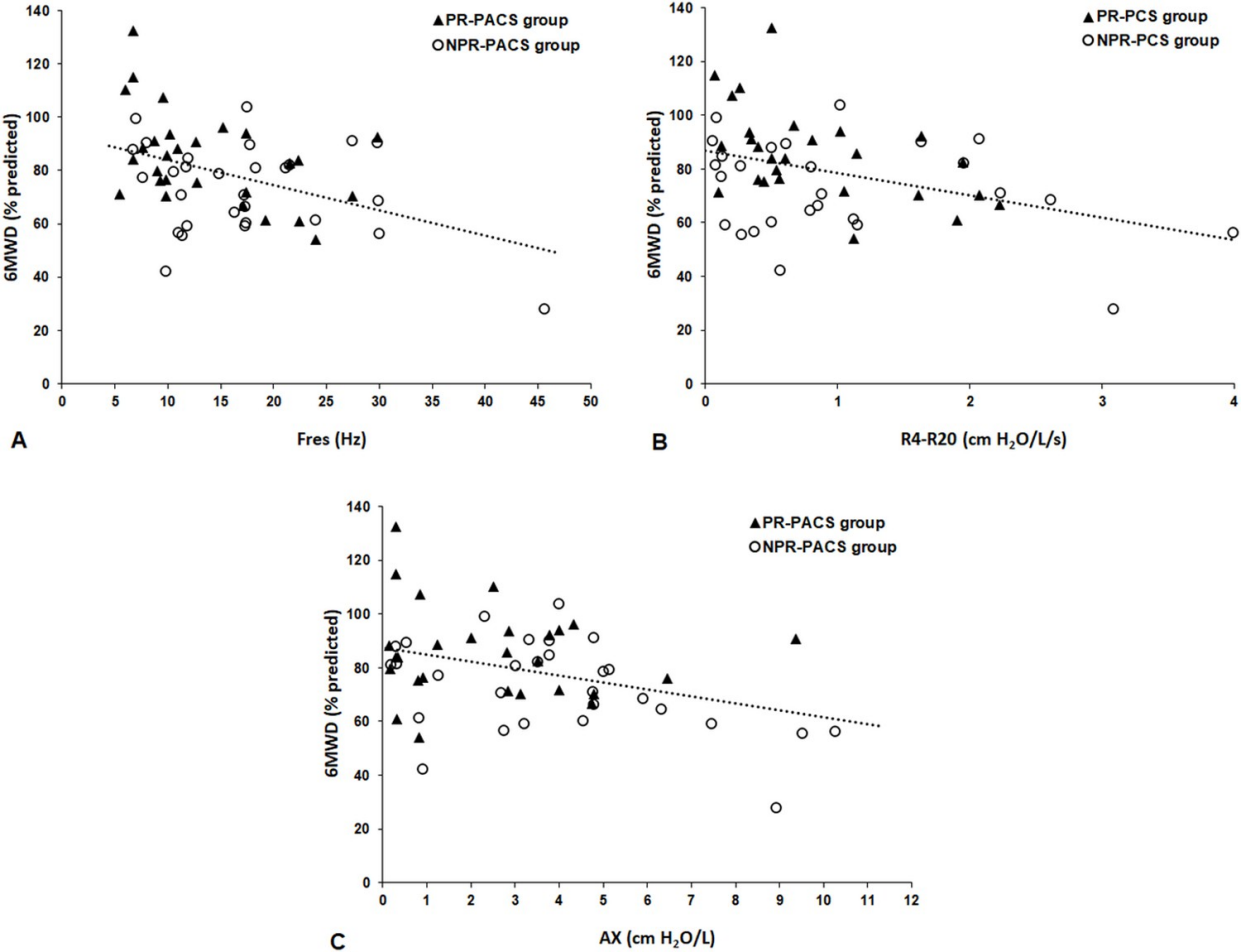
Relationships of the 6MWD with Fres (r = -0.34, p = 0.009) (A), R4-R20 (r = -0.34, p = 0.010) (B) and AX (r_s_ = -0.30, p = 0.026) (C). 6MWD = 6-min walk distance; Fres = resonance frequency; R4-R20 = heterogeneity of resistance between R4 and R20; AX = area under the reactance curve; PR-PACS = patients with post-COVID syndrome who underwent pulmonary rehabilitation; NPR-PACS = patients with post-COVID syndrome who did not undergo pulmonary rehabilitation.

The associations of 6MWD (% predicted) with pulmonary function parameters and LUS signs are shown in Table 4 and Fig 1. In the PR-PACS group, 6MWD correlated significantly with Fres, Rm, R4, R20 and R4-R20. In the PR-PACS group, 6MWD correlated significantly with Fres, Rm, R4, R20 and R4-R20. In the NPR-PACS group, 6MWD correlated significantly with AX, end-of-test HR, resting VE, VE_peak_, ΔIC (L) and ΔIC (%). In the PR-PACS group, patients with abnormal IOS walked less in the 6MWT than patients with normal IOS [74 ± 14.5 vs. 88.9 ± 17.1% predicted, p = 0.040]. There was no association between abnormal spirometry and 6MWD or between abnormal LUS and 6MWD.

**Table 4:** Correlation coefficients for six‐minute walk test, pulmonary function parameters and lung ultrasound signals.

Variable	6MWD (% predicted)					
	Total sample		PR-PACS group		NPR-PACS group	
	r	p-value	R	p-value	r	p-value
**Resting variables**						
FVC	0.13	0.30	0.16	0.38	0.14	0.44
FEV_1_	0.03	0.79	-0.08	0.65	0.16	0.39
FEV_1_/FVC	0.03	0.83	-0.13	0.47	-0.01	0.96
FEF_25-75%_	0.19	0.12	0.17	0.33	0.22	0.22
Fres	**-0.34**	**0.009**	**-0.44**	**0.02**	-0.09	0.64
Rm	-0.27	0.09	**-0.47**	**0.011**	-0.05	0.81
R4	**-0.34**	**0.008**	**-0.51**	**0.005**	-0.19	0.32
R20	**-0.32**	**0.014**	**-0.43**	**0.025**	-0.24	0.22
R4-R20	**-0.34**	**0.010**	**-0.56**	**0.002**	-0.17	0.37
AX	**-0.30**	**0.026**	-0.13	0.52	**-0.38**	**0.044**
Aeration score	-0.13	0.32	0.03	0.88	-0.12	0.50
**Exercise variables**						
Basal SpO_2_	-0.03	0.83	-0.06	0.76	-0.15	0.42
End-of-test SpO_2_	-0.01	0.99	0.05	0.77	-0.15	0.41
Basal HR	-0.01	0.94	0.30	0.09	-0.09	0.62
End-of-test HR	**0.30**	**0.014**	0.23	0.21	**0.53**	**0.002**
Resting VE	**0.25**	**0.044**	-0.11	0.53	**0.46**	**0.008**
VE_peak_	0.19	0.14	0.02	0.94	**0.48**	**0.005**
BR	-0.04	0.73	0.22	0.21	-0.29	0.10
Basal IC	-0.10	0.43	-0.25	0.15	-0.13	0.49
End-of-test IC	-0.13	0.31	-0.27	0.13	-0.15	0.41
ΔIC (L)	0.06	0.61	0.03	0.86	**-0.37**	**0.038**
ΔIC (%)	0.08	0.54	0.18	0.31	**-0.41**	**0.018**

6MWD = six‐minute walking distance; PR-PACS = patients with post-COVID syndrome who underwent pulmonary rehabilitation; NPR-PACS = patients with post-COVID syndrome who did not undergo pulmonary rehabilitation; FEF_25-75%_ = forced expiratory flow during the middle half of the FVC maneuver; Fres = resonance frequency; Rm = mean resistance between 4–20 Hz; R4 = resistance at 4 Hz; R20 = resistance at 20 Hz; R4-R20 = heterogeneity of resistance between R4 and R20; AX = area under the reactance curve; SpO_2_ = oxygen saturation; HR = heart rate; VE = minute ventilation; BR = breathing reserve; IC = inspiratory capacity.

## Discussion

There are few studies investigating the efficacy of PR in PACS, with the vast majority of them focusing on the acute phase of COVID-19 [13]. With this in mind, we sought to evaluate PACS outpatients, as this subgroup accounts for the vast majority of the post-COVID-19 population. The main findings of the present study were that PACS patients undergoing PR had better performance on the 6MWT than PACS patients not undergoing PR, with a higher 6MWD and less DH. PACS patients undergoing PR had less abnormal IOS than PACS patients not undergoing PR, although these groups were not differentiated by spirometry or LUS. The more preserved the respiratory system mechanics assessed by IOS, the better the performance on the 6MWT in PACS patients. Furthermore, there was a relationship between DH and worse performance in the 6MWT. To the best of our knowledge, this is the first study to evaluate the effects of PR using the ventilatory dynamics measured during the Spiropalm^®^-equipped 6MWT in individuals with PACS.

The 6MWT is recognized as a simple, efficient and low-cost tool that allows the evaluation of patient performance and cardiopulmonary fitness during submaximal exercise and is routinely used in clinical research as the main primary endpoint to evaluate the benefits of PR [23]. In our study, PACS patients undergoing PR had a higher 6MWD than PACS patients not undergoing PR. In line with our findings, Nopp et al. [22] and Araújo et al. [37] observed improvement in submaximal exercise tolerance in PACS patients, including a minimum clinically important difference in 6MWD. Unlike our sample that underwent PR, however, these two studies included both hospitalized and ICU patients, which makes it difficult to exclude the impact of changes inherent to critically ill patients, such as prolonged immobility, the use of sedatives related to muscle weakness and the damage caused by mechanical ventilation [37]. Despite the discrepancies in the sample characteristics of the various studies evaluating PR in PACS, it is increasingly clear that PR may be a valuable treatment option in patients with PACS, even in those individuals who do not require hospitalization. The PR aims to revert or minimize the sequelae from the acute disease phase, improve the QoL and return the individual to the lifestyle he had before the disease. In general, and considering the main symptoms observed, this process includes a decrease in dyspnea and general fatigue and an increase in strength, resistance and exercise tolerance with a consequent return to ADLs and social reintegration [6, 38]. It is possible that the increased ventilation provided by physical training affects the respiratory muscles, increasing oxidative fibers and the activity of oxidative enzymes [39].

HD is a strong determinant of exercise tolerance because it increases the mechanical load on the inspiratory muscles and impairs the ability of tidal volume to increase adequately with exercise. Using IC to estimate DH, we observed that 37.2% of the individuals in the NPR-PACS group had DH during the 6MWT, whereas this was observed only in 6.1% of the patients in the PR-PACS group, with significant differences between these two groups relative to the ΔIC. In addition to being an indirect estimate of the degree of hyperinflation, IC is physiologically determined by the inspiratory muscle strength and extent of the intrinsic mechanical load on the inspiratory muscles [20]. Using CPET in a sample of patients with severe COVID-19, Noureddine et al. [5] observed that although only a minority of patients had significant impairment of respiratory function at rest (as was the case of the patients in our study), more than half of the patients with normal VO_2peak_ had ventilatory inefficiency during exercise, with an abnormal increase in physiological dead space ventilation and a wide alveolar-arterial gradient. In the study by Noureddine et al. [5], 80% of the individuals underwent PR, although the authors did not directly assess its impact. Importantly, the VO_2peak_ was strongly correlated with 6MWT, indicating the importance of using the 6MWT in monitoring this population. Of note, we found significant correlations between the measurements of VE and ΔIC with 6MWD in patients in the NPR-PACS group. Because it is able to combine alveolar ventilation with metabolic rate, VE increases during exercise, and consequently, VE_peak_ is one of the main determinants of exercise tolerance, which may explain the relationship between VE_peak_ and 6MWD.

In our study, resting lung function assessed by traditional PFTs was little altered in either the PR-PACS or the NPR-PACS groups, which suggests that these measures are not ideal for assessing the impact of a PR program. In fact, the effects of different rehabilitative strategies on lung function in patients with PACS are still controversial, with some studies showing the efficacy of these programs in improving muscle function and QoL but without any impact on lung function [17, 18]. In this scenario, IOS may be particularly beneficial for identifying subtle changes in lung function after COVID-19 [35]. In fact, we observed that patients in the PR-PACS group had fewer IOS abnormalities than those in the NPR-PACS group, which indicates the importance of IOS as a complementary method to traditional PFTs. A possible explanation for the greater sensitivity of IOS in detecting the effects of PR is that it assesses the total behavior of the respiratory system (including the airways, lung tissue and chest wall) [40], and PR can greatly affect the respiratory muscle function of these patients with improvement of the reactive and resistive properties of the respiratory system.

In the present study, we observed significant correlations between several parameters measured by the IOS (including those that detect early changes in small airways) and the 6MWD. In line with our findings, Bonato et al. [41] found that persistent dyspnea in PACS (which can be relieved by an PR program) is associated with small airway dysfunction 3 months after discharge, regardless of the parenchymal/radiological sequelae. A follow-up observational study of PACS individuals showed that, in the multiple regression analysis, reactance at 4 Hz, forced expiratory flow during the middle half of the FVC maneuver and Fres were significantly related to the severity of COVID-19 [42]. We believe that it is likely that the improvement in respiratory mechanics of these patients with PR, especially peripheral and tissue resistance (chest wall), may increase exercise tolerance with a positive impact on functional independence and QoL. Finally, it is noteworthy that no difference was observed in the LUS signals between the PR-PACS and NPR-PACS groups, which indicates that the structural changes of the PACS are not influenced by the PR.

Some limiting aspects need to be considered when interpreting the findings of our study. First, no causal role of PR can be assumed with certainty due to our observational study design. Second, we do not have data prior to PR because it is a cross-sectional study, although conducting a randomized controlled trial on the effects of PR may be considered unethical due to a lack of clinical balance [43]. Third, we did not use measures of clinical outcome that were demonstrably effective in individuals with PACS, such as diffusing capacity for carbon monoxide and computed tomography, although our intention was to focus on simpler techniques to assess outcomes. Despite these limitations, we believe that our study may serve as a starting point for incorporating the dynamics of ventilation during the 6MWT and IOS in the evaluation of PR. Future PR studies may shed more light on the optimal treatment of patients with PACS and the use of more effective measures to assess the endpoints, taking into account cost-effectiveness. It is necessary to bear in mind the need for a multidisciplinary approach due to the multisystemic nature of PACS, and the recommended PR intensity must take into account the patient’s tolerance for training execution and progression [6, 38].

In conclusion, our study shows that PR in PACS patients improves the functional capacity assessed by the 6MWT, with a higher 6MWD and less HD. In these patients, IOS is able to distinguish the effects of PR that are not differentiated by spirometry or LUS. In these patients, there was a relationship between respiratory mechanics assessed by IOS and performance on the 6MWT. Furthermore, there is an association between DH assessed during the 6MWT and worse performance in the 6MWT. With this in mind, we think that our findings may provide information for outcome measures in PR programs in patients with PACS.

## Supporting information

S1 Checklist STROBE statement—Checklist of items that should be included in reports of observational studies. (PDF)
